# Supplementary material for: The Systems Biology Research Tool: evolvable open-source software
Source: BMC Syst Biol. 2008 Jun 29;2:55. doi: 10.1186/1752-0509-2-55 (PMC2446383; doi:10.1186/1752-0509-2-55)
Supplement: Additional file 1 — SBRT Archive. An archive of the current version of the Systems Biology Research Tool. [file 1752-0509-2-55-S1.zip › sbrt-1.4.0/doc/developers_guide/api/sbrt/kernel/fba/AbstractRxn.html]

AbstractRxn


|  |  |  |
| --- | --- | --- |
| **Overview** \| **Package** \| **Class** \| **Use** \| **Tree** \| **Deprecated** \| **Index** \| **Help** | |  |
| PREV CLASS   **NEXT CLASS** | **FRAMES**    **NO FRAMES**     **All Classes** |  |
| SUMMARY: NESTED \| FIELD \| CONSTR \| METHOD | DETAIL: FIELD \| CONSTR \| METHOD |  |


---


## sbrt.kernel.fba Class AbstractRxn

```
java.lang.Object
  sbrt.kernel.fba.AbstractRxn
```

**All Implemented Interfaces:**: ChemRxn

**Direct Known Subclasses:**: IrrevRxn, ReversibleRxn

---

``` public abstract class AbstractRxn extends java.lang.Object implements ChemRxn ```

This class is a skeleton implementation of ChemicalRxn. It performs basic
functions that are useful for most classes implementing ChemicalRxn.

**Author:**
:   This class was written and documented by Jeremiah Wright while in the
    Wagner lab.

---

**Constructor Summary**

`AbstractRxn(LinearComb<java.lang.String> reactants,
LinearComb<java.lang.String> products)`
  
          Constructs a new chemical reaction from the provided reactants
and products with the given name.


| **Method Summary** | |
| --- | --- |
| `boolean` | `equals(java.lang.Object obj)`              Indicates if the provided object is equal to this chemical reaction. |
| `LinearComb<java.lang.String>` | `getProducts()`             Returns the products of this reaction. |
| `protected  double` | `getRatio(LinearComb<?> expr1, LinearComb<?> expr2)`             Returns the ratio of the coefficients in the provided expressions. |
| `LinearComb<java.lang.String>` | `getReactants()`             Returns the reactants of this reaction. |
| `int` | `hashCode()`             Returns a content-based hash code for this reaction. |
| `static void` | `main(java.lang.String[] args)`             Used for testing purposes. |
| `abstract  java.lang.String` | `toString()`             Returns an appropriate string representation of this reaction. |

**Methods inherited from class java.lang.Object**

`clone, finalize, getClass, notify, notifyAll, wait, wait, wait`

**Methods inherited from interface sbrt.kernel.fba.ChemRxn**

`isEquivalent, isReverse`

**Constructor Detail**

### AbstractRxn

```
public AbstractRxn(LinearComb<java.lang.String> reactants,
                   LinearComb<java.lang.String> products)
```

:   Constructs a new chemical reaction from the provided reactants
    and products with the given name.

    **Parameters:**: `reactants` - the linear combination of chemical species representing the "left-hand side" of the reaction.: `products` - the linear combination of chemical species representing the "right-hand side" of the reaction. **Throws:**: `java.lang.NullPointerException` - if the reactants or products are `null`, or if any chemical species within them is `null`.: `java.lang.IllegalArgumentException` - if • the reactants or products contain a common chemical species, • either linear combination contains a non-zero constant, • a stoichiometric coefficient is NaN, infinite, or ≤ 0.


**Method Detail**

### getReactants

```
public final LinearComb<java.lang.String> getReactants()
```

:   Returns the reactants of this reaction.

    :   **Specified by:**: `getReactants` in interface `ChemRxn`
    :   **Returns:**: the reactants of this reaction.

---


### getProducts

```
public final LinearComb<java.lang.String> getProducts()
```

:   Returns the products of this reaction.

    :   **Specified by:**: `getProducts` in interface `ChemRxn`
    :   **Returns:**: the products of this reaction.

---


### toString

```
public abstract java.lang.String toString()
```

:   Returns an appropriate string representation of this reaction.

    :   **Specified by:**: `toString` in interface `ChemRxn` **Overrides:**: `toString` in class `java.lang.Object`
    :   **Returns:**: an appropriate string representation of this reaction.

---


### hashCode

```
public int hashCode()
```

:   Returns a content-based hash code for this reaction.

    :   **Specified by:**: `hashCode` in interface `ChemRxn` **Overrides:**: `hashCode` in class `java.lang.Object`
    :   **Returns:**: a content-based hash code for this reaction.

---


### equals

```
public boolean equals(java.lang.Object obj)
```

:   Indicates if the provided object is equal to this chemical reaction.

    For a reaction `r` to be equal to this reaction,
    `isEquivalent(r)` must return `true` and both reactions must
    have the same name and catalyst.

    :   **Specified by:**: `equals` in interface `ChemRxn` **Overrides:**: `equals` in class `java.lang.Object`
    :   **Parameters:**: `obj` - the reference object with which to compare. **Returns:**: `true` if this reaction is equal to the provided provided object; `false` otherwise. **See Also:**: `ChemRxn.isEquivalent(ChemRxn)`

---


### getRatio

```
protected double getRatio(LinearComb<?> expr1,
                          LinearComb<?> expr2)
```

:   Returns the ratio of the coefficients in the provided expressions.

    :   **Parameters:**: `expr1` - a stoichiometric expression.: `expr2` - a stoichiometric expression. **Returns:**: the coefficients of `expr1` divided by the coefficients of `expr2`; or `Double.NaN` if the coefficients of the terms in the provided expressions are not factors of each other.

---


### main

```
public static void main(java.lang.String[] args)
```

:   Used for testing purposes.

    :   **Parameters:**: `args` - ignored.


---


|  |  |  |
| --- | --- | --- |
| **Overview** \| **Package** \| **Class** \| **Use** \| **Tree** \| **Deprecated** \| **Index** \| **Help** | |  |
| PREV CLASS   **NEXT CLASS** | **FRAMES**    **NO FRAMES**     **All Classes** |  |
| SUMMARY: NESTED \| FIELD \| CONSTR \| METHOD | DETAIL: FIELD \| CONSTR \| METHOD |  |


---
